# Cuproptosis: a novel mechanism linking placental metabolic stress to trophoblast injury in preeclampsia

**DOI:** 10.3389/fendo.2026.1926486

**Published:** 2026-09-09

**Authors:** Ling Liu, Baoxiang Xing, Yan Zhang

**Affiliations:** The Affiliated Hospital of Qingdao University, Qingdao, China

**Keywords:** copper homeostasis, cuproptosis, placental hypoxia, preeclampsia, trophoblast injury

## Abstract

Preeclampsia (PE) is characterized by placental hypoxia, metabolic stress, trophoblast dysfunction, and impaired spiral artery remodeling, but the mechanisms linking these abnormalities remain incompletely understood. Cuproptosis, a recently identified form of regulated cell death driven by copper-dependent aggregation of lipoylated tricarboxylic acid cycle proteins, may provide a mechanistic link between placental metabolic stress and trophoblast injury. Here, we propose a hypothesis in which hypoxia-induced glycolytic reprogramming and lactate accumulation promote histone H3K18 lactylation, activate GRHL2, and increase SLC31A1/CTR1-mediated copper uptake. Subsequent FDX1-dependent copper reduction, DLAT aggregation, Fe-S cluster loss, and mitochondrial proteotoxic stress may preferentially affect mitochondria-rich villous syncytiotrophoblasts, while also impairing EVT function. Cuproptosis may interact with ferroptosis and oxidative stress through shared metal-redox and mitochondrial vulnerabilities. Although current evidence does not establish cuproptosis as an initiating cause of PE, it may amplify placental dysfunction. Targeting copper transport, mitochondrial stress, or pathway-specific biomarkers warrants further investigation.

## 1 Introduction

Preeclampsia (PE) affects approximately 2–8% of pregnancies worldwide and remains one of the leading causes of maternal and perinatal morbidity and mortality. The core pathology resides in defective extravillous trophoblast (EVT) invasion and inadequate spiral artery remodeling, leading to reduced utero-placental perfusion and placental hypoxia, which subsequently triggers oxidative stress and an elevated soluble fms-like tyrosine kinase-1 (sFlt-1)/placental growth factor (PlGF) ratio (1). However, the precise molecular mechanisms that translate placental metabolic stress into trophoblast injury remain incompletely elucidated.

Cuproptosis is a recently identified form of regulated cell death first described by Tsvetkov et al. in 2022 (2). Its mechanism is distinct: excess intracellular copper (Cu²⁺) is reduced to the more toxic Cu⁺ form by ferredoxin 1 (FDX1); Cu⁺ then directly binds to lipoylated components of the tricarboxylic acid (TCA) cycle, most notably dihydrolipoamide S-acetyltransferase (DLAT), triggering its oligomerization and aggregation, followed by loss of iron-sulfur (Fe-S) cluster proteins and mitochondrial proteotoxic collapse. Unlike apoptosis and ferroptosis, cuproptosis is independent of caspase activation and lipid peroxidation (2). Notably, cells dependent on oxidative phosphorylation are approximately 1,000-fold more sensitive to cuproptosis than glycolytic cells (2), and syncytiotrophoblasts are precisely such a highly metabolic, mitochondria-dependent cell type, suggesting they may represent a natural susceptibility target for cuproptosis.

Cellular susceptibility is also likely heterogeneous. Syncytiotrophoblasts, which line chorionic villi and are directly exposed to maternal blood, are mitochondria-rich and therefore may be especially vulnerable to DLAT-centered cuproptosis; EVTs at the decidual spiral-artery interface may be less intrinsically oxidative but may undergo secondary migratory and invasive dysfunction when hypoxia-lactate signaling and altered copper handling converge.

## 2 Cuproptosis and preeclampsia: emerging direct evidence

In recent years, multiple clinical studies have provided evidence for copper dyshomeostasis in PE. Gul et al. conducted a case-control study of 88 third-trimester pregnant women and found significantly elevated serum copper levels (predictive cut-off: 224 μg/dL) and decreased thiol levels in PE patients, with the copper/native thiol ratio demonstrating predictive value (3). Zhong et al.’s systematic review and meta-analysis of 34 studies (2,471 PE cases vs. 2,888 controls) revealed a more complex picture: the association between maternal serum copper levels and PE exhibits significant geographic variation, with a potential J-shaped relationship (4). Intriguingly, Hao et al. reached a contrasting conclusion at the placental tissue level—copper, zinc, selenium, and other metals were significantly reduced in PE placentas (5), suggesting that placental copper transport dysregulation, rather than simple systemic copper excess, may represent the operative pathology.

Two recent studies have directly linked cuproptosis to PE. Tang et al. analyzed transcriptomic data from PE and normal placental tissues, identifying five differentially expressed cuproptosis-related genes:NFE2L2, PDHA1, PDHB, DLD, and GLS, among which NFE2L2 was identified as the key hub gene (6). More importantly, Shen et al. demonstrated mechanistic evidence that lactate accumulated in PE placentas activates the transcription factor Grhl2 through histone H3K18 lactylation (H3K18la), which in turn transcriptionally upregulates the copper transporter SLC31A1 (CTR1), leading to intracellular copper overload and trophoblast cuproptosis; knockdown of SLC31A1 blocked cuproptosis and rescued trophoblast function (7). This landmark study is the first to integrate metabolic dysregulation, epigenetic reprogramming, copper transport derangement, and trophoblast cuproptosis into one coherent causal chain.

These data should be interpreted with caution in relation to disease timing. Total serum copper does not necessarily reflect the labile copper pool available to trophoblast mitochondria, because circulating copper is largely carried by ceruloplasmin, a major serum copper transport protein and positive acute-phase response protein (8, 9). Thus, PE-associated inflammation and socioeconomic/ethnic differences in nutritional status may confound systemic copper measurements (4). We therefore propose cuproptosis as a stress-amplifying and potentially causal module within placental dysfunction, but not yet as a proven initiating event of PE; temporal studies will be needed to distinguish whether copper-driven trophoblast death precedes, accompanies, or follows the inflammatory-metabolic syndrome.

## 3 From hypoxia to copper overload: an integrative pathway

**Figure 1:**
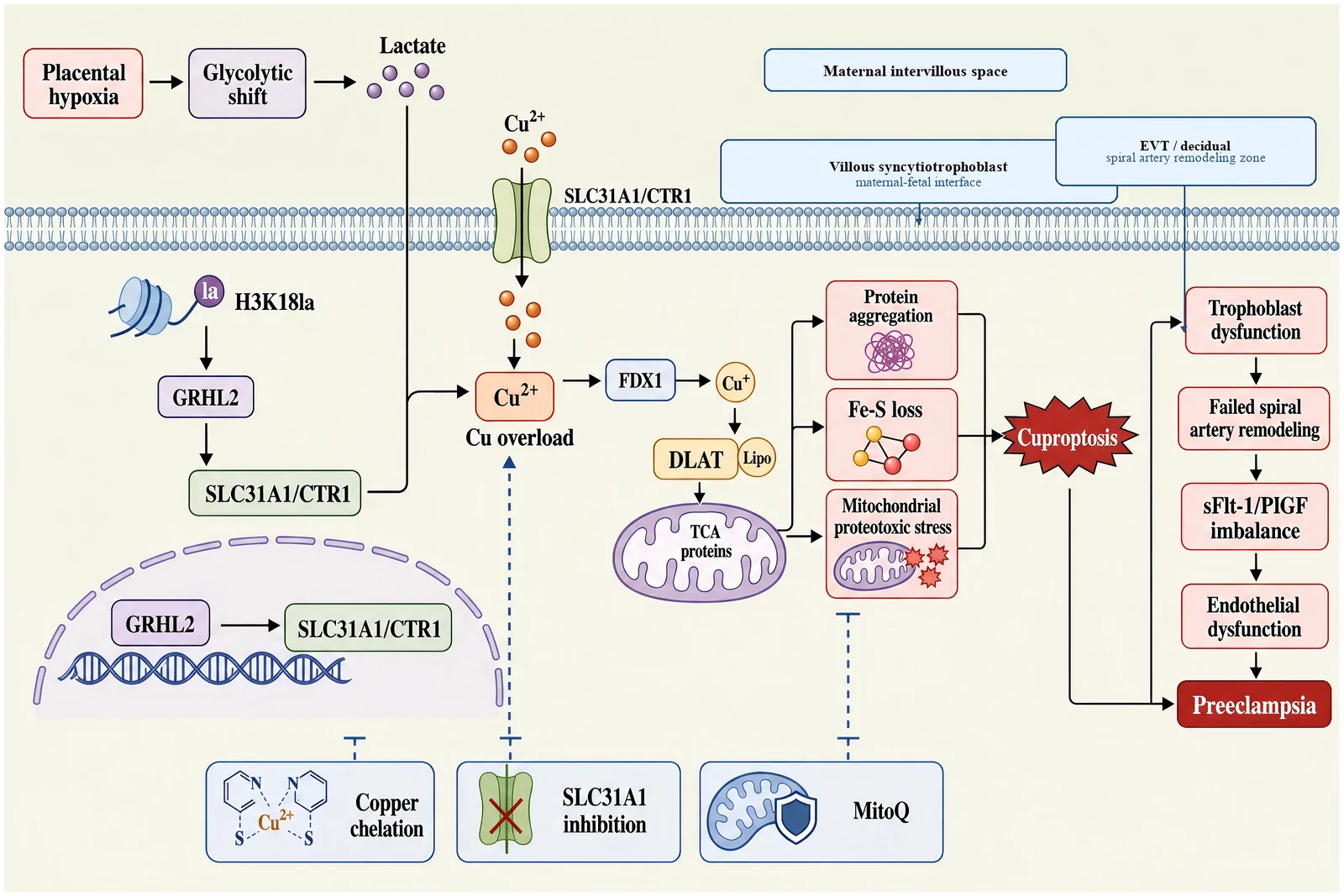
Schematic illustration of the proposed mechanism of cuproptosis in preeclampsia and potential interventional targets. Under placental hypoxia characteristic of PE, enhanced glycolysis leads to lactate accumulation, which induces histone H3K18 lactylation (H3K18la) and GRHL2 activation, upregulating SLC31A1/CTR1 to promote copper uptake and intracellular copper overload. FDX1-mediated Cu2+ reduction and subsequent Cu+ binding to lipoylated DLAT trigger protein aggregation, Fe-S cluster loss, and mitochondrial proteotoxic stress, culminating in trophoblast cuproptosis. Cuproptosis-driven trophoblast impairment and failed spiral artery remodeling exacerbate placental hypoxia, promoting sFlt-1/PlGF imbalance, endothelial dysfunction, and gestational hypertension that drive PE progression. Copper chelators, SLC31A1 inhibitors, and mitochondrial antioxidants such as MitoQ may confer protection by attenuating copper overload and mitochondrial oxidative injury.Solid arrows indicate promoting or progressive relationships; dashed arrows indicate inhibitory or protective effects. Histologically, this cascade is proposed to operate mainly in the villous syncytiotrophoblast layer at the maternal-fetal interface, with secondary effects on EVT function in decidual spiral-artery remodeling zones.

Integrating the above evidence, we propose the following hypothetical pathway: placental hypoxia (secondary to defective spiral artery remodeling) drives syncytiotrophoblasts to enhance anaerobic glycolysis, resulting in substantial lactate accumulation. Lactate functions not merely as a metabolic waste product but as an epigenetic substrate (**Figure 1**). Li et al. first demonstrated that hypoxia upregulates fibrosis-related genes FN1 and SERPINE1 in PE placentas via histone lactylation (10), and subsequently discovered that lactate promotes premature placental senescence through H3K18la modification of the GADD45A promoter (11). Shen et al.’s study (7) further extended this epigenetic regulatory mechanism to the copper metabolism domain: under hypoxia-induced lactate accumulation, H3K18la upregulates GRHL2 expression, which in turn promotes SLC31A1 transcription, enhances cellular copper uptake, and ultimately triggers cuproptosis through the FDX1/DLAT-mediated pathway. This establishes an integrated cascade linking placental hypoxia, via lactate-driven epigenetic reprogramming, to copper overload and cuproptosis.

Concurrently, Opichka et al. employed Cre-lox and DREADD technology to selectively activate Gαq signaling in mouse syncytiotrophoblasts, demonstrating that syncytiotrophoblast mitochondrial oxidative stress is sufficient to drive PE-like phenotypes, including hypertension, proteinuria, and reduced placental vascularization, which were significantly attenuated by the mitochondrial-targeted antioxidant MitoQ (12). Given that the core execution site of cuproptosis is precisely the mitochondrion, and that cells with active mitochondrial metabolism are most vulnerable to cuproptosis (2), these findings provide indirect yet compelling support for the involvement of cuproptosis in PE pathogenesis.

Conceptually, cuproptosis may also intersect with ferroptosis and generalized oxidative stress in PE. Recent work shows that placental hypoxia and iron overload can induce trophoblast ferroptosis and vascular injury in PE (13, 14). Although cuproptosis is executed through lipoylated-protein aggregation rather than lipid peroxidation, DLAT aggregation and Fe-S cluster loss may impair mitochondrial electron transport, increase reactive oxygen species, and weaken glutathione/GPX4-dependent antioxidant buffering, thereby lowering the threshold for iron-dependent lipid peroxidation. Accordingly, cuproptosis and ferroptosis may represent connected outputs of a shared metal-metabolism-redox vulnerability state rather than mutually exclusive mechanisms (15).

## 4 Translational implications and future directions

The cuproptosis hypothesis offers novel therapeutic entry points for PE. Blocking aberrant copper influx is the first potential strategy. Shen et al. have already demonstrated that targeting SLC31A1 inhibits cuproptosis and ameliorates trophoblast dysfunction (7). Mitochondrial protection represents the second strategy: the efficacy of MitoQ in animal models provides a rationale for further exploring mitochondrially targeted antioxidants in PE (12). A third potential avenue is the direct modulation of key molecules in the cuproptosis pathway, such as FDX1 or DLAT (2). However, interventional strategies during pregnancy face unique challenges: copper is an essential trace element for fetal development, and systemic copper chelation may precipitate fetal copper deficiency. Therefore, placenta-specific delivery systems or selective SLC31A1 inhibitors may carry greater clinical feasibility than broad-spectrum copper chelators.

It must be emphasized, however, that direct evidence supporting a causal role for cuproptosis in PE pathogenesis remains limited at present. Priority areas for future investigation include: (i) direct detection of cuproptosis markers in PE placental tissues; (ii) functional validation of the causal relationship between copper overload and cuproptosis using primary trophoblasts and placental organoids; (iii) generation of placenta-specific SLC31A1 or FDX1 conditional knockout animal models to evaluate causal effects on PE phenotypes; and (iv) prospective cohort studies to assess the value of cuproptosis-related molecules as early predictive biomarkers for PE. Should future studies confirm its pathogenic significance, cuproptosis may open new avenues for both mechanistic understanding and targeted intervention in preeclampsia.

In this context, immunohistochemical or multiplex immunofluorescent mapping of FDX1 and DLAT expression intensity, lipoylated-DLAT aggregation, and their cellular localization in villous syncytiotrophoblasts versus EVTs could help determine whether these molecules have biomarker value and clarify the histological compartment in which cuproptosis is most active.

## Data Availability

The original contributions presented in the study are included in the article/supplementary material. Further inquiries can be directed to the corresponding author.
